# Integrated Immune–Gut Profiling Identifies an Exploratory Pediatric Inflammatory Intestinal Profile Associated with Food-Specific IgG Reactivity

**DOI:** 10.3390/biom16060922

**Published:** 2026-06-22

**Authors:** Laura-Mihaela Ion, Carmen Pavelescu, Denisa Maria Canut, Mihaela Oros, Gheorghita Jugulete, Smaranda Diaconescu

**Affiliations:** 1 Department of Pediatrics, University of Medicine Titu Maiorescu, 040441 Bucharest, Romania; 2 Ponderas Academic Hospital, No. 85A, Nicolae G. Caramfil Street, 014142 Bucharest, Romania; 3 Department of Infectious Diseases, University of Medicine and Pharmacy “Carol Davila”, No. 37, Dionisie Lupu Street, 2nd District, 020021 Bucharest, Romania; 4 Department of Physiology, Faculty of Medicine, Titu Maiorescu University, No. 67A Gheorghe Petrascu Street, 3rd District, 031593 Bucharest, Romania; 5 “Matei Balş” National Institute for Infectious Diseases, No. 1, Calistrat Grozovici Street, 2nd District, 021105 Bucharest, Romania

**Keywords:** food-specific IgG, pediatric gastrointestinal disorders, ultrasound, bowel wall thickening, intestinal inflammatory biomarkers, non-IgE hypersensitivity, food intolerance

## Abstract

The clinical relevance of food-specific IgG antibodies in pediatric gastrointestinal disorders remains controversial. Although current international guidelines discourage their use as standalone diagnostic tools, their significance within a broader immune–gut inflammatory framework has not been sufficiently explored. This study aimed to investigate associations between food-specific IgG reactivity, inflammatory and permeability biomarkers, microbiological findings, and abdominal ultrasound abnormalities in children with chronic gastrointestinal symptoms. Methods: (1) Children presenting chronic gastrointestinal symptoms associated with food-specific IgG polysensitization, elevated inflammatory and permeability biomarkers, and abdominal ultrasound abnormalities (number (n) = 196); (2) a symptomatic gastrointestinal group without the complete multimodal profile (n = 146); and (3) a control group with normal abdominal ultrasound findings and biomarkers within reference ranges (n = 210). All participants underwent food-specific IgG testing using a 216-antigen ELISA panel, abdominal ultrasound examination, and assessment of intestinal inflammatory and permeability biomarkers. Food-specific IgG antibodies were not interpreted as diagnostic markers of food allergy or food intolerance. Comparative analyses, correlation analyses, multivariable logistic regression, and receiver operating characteristic (ROC) analyses were performed. Results: Food-specific IgG polysensitization was significantly more frequent among children presenting the multimodal inflammatory profile compared with symptomatic and control groups (all *p* < 0.001). Reactivity predominantly involved gluten-containing cereals, dairy proteins, and mixed gluten–dairy patterns. Elevated fecal calprotectin, zonulin, and fecal histamine concentrations were more frequently observed in this subgroup, together with a higher prevalence of ultrasound abnormalities, including bowel wall thickening and mesenteric lymphadenopathy. Correlation analyses demonstrated significant associations between cumulative IgG burden and bowel wall thickness (r = 0.48, *p* < 0.001), while fecal calprotectin showed the strongest association with ultrasound abnormalities (r = 0.62, *p* < 0.0001). Multivariable logistic regression identified elevated calprotectin, increased zonulin, IgG polysensitization, and mixed gluten–dairy reactivity as independent predictors of pathological ultrasound findings. The integrated multimodal model demonstrated higher classification performance than isolated biomarkers. Conclusions: Children presenting chronic gastrointestinal symptoms, food-specific IgG polysensitization, inflammatory biomarker abnormalities, and ultrasound changes represented a multimodal clinical subgroup within the study population. These findings support evaluating food-specific IgG reactivity within a broader immune–gut assessment framework rather than as a standalone diagnostic biomarker. The observed associations should be considered exploratory and hypothesis-generating, requiring prospective validation and mechanistic investigation.

## 1. Introduction

Functional gastrointestinal disorders (FGIDs) and non-IgE-mediated food-related hypersensitivity reactions are increasingly recognized as important contributors to pediatric morbidity and reduced quality of life [1,2]. A substantial proportion of children present with persistent gastrointestinal manifestations, including recurrent abdominal pain, bloating, altered bowel habits, nausea, food-related symptoms, and extra-intestinal complaints, despite the absence of overt structural gastrointestinal disease [3]. This persistent discrepancy between symptom burden and the lack of conventional organic pathology highlights a major diagnostic and pathophysiological gap in pediatric gastroenterology.

Recent advances in mucosal immunology and microbiome research suggest that low-grade intestinal inflammation, intestinal barrier dysfunction, chronic antigen exposure, and microbiota-related immune activation may contribute significantly to pediatric gastrointestinal symptomatology [4,5]. The emerging concept of the “immune–gut axis” posits that those interactions among dietary antigens, epithelial permeability, the gut microbiota, and host immune responses may lead to chronic immune-mediated intestinal dysfunction, even in the absence of classical inflammatory bowel disease [6].

Within this context, food-specific immunoglobulin G (IgG) antibodies remain a highly controversial topic in both allergy and gastroenterology [7]. Unlike IgE-mediated food allergies, which are characterized by immediate hypersensitivity reactions and well-established pathogenic mechanisms, IgG-mediated responses are typically delayed, nonspecific, and difficult to interpret clinically [8]. Consequently, several international scientific societies discourage the use of food-specific IgG testing as a standalone diagnostic method for food allergy or intolerance, emphasizing that IgG antibodies may primarily represent physiological exposure and immunological tolerance [9,10].

Nevertheless, increasing evidence suggests that food-specific IgG responses may acquire clinical significance when associated with broader biological alterations, including increased intestinal permeability, chronic antigenic stimulation, microbiome dysregulation, and low-grade inflammatory activation [11,12]. In pediatric populations, this issue is particularly relevant because the developing immune system interacts dynamically with dietary antigens and intestinal microbiota during critical periods of immune maturation [13].

Several studies have reported associations between food-specific IgG positivity and conditions such as irritable bowel syndrome (IBS), migraine, eczema, chronic fatigue, inflammatory bowel disease, and functional gastrointestinal disorders [14,15,16,17]. Moreover, some clinical studies suggest that elimination diets guided by food-specific IgG panels may improve symptoms in selected patient subgroups [18,19]. However, the underlying biological mechanisms remain poorly defined, and most previous investigations have relied predominantly on symptom-based outcomes rather than objective multimodal biomarkers.

Among the most relevant biomarkers of intestinal dysfunction, fecal calprotectin has emerged as a sensitive indicator of mucosal inflammation, while zonulin has been increasingly investigated as a regulator of intestinal epithelial tight junctions and intestinal permeability [20,21,22]. Elevated fecal histamine may reflect mast-cell-associated mucosal activity and immune-mediated mucosal responses [23]. Simultaneously, growing evidence suggests that microbiological factors, including protozoal organisms such as Dientamoeba fragilis, may contribute to chronic gastrointestinal symptoms, intestinal immune activation, and low-grade inflammatory changes in children [24,25].

Abdominal ultrasound has become an increasingly valuable non-invasive imaging modality in pediatric gastroenterology [26]. Beyond excluding acute surgical or inflammatory conditions such as appendicitis, intussusception, or Crohn’s disease, intestinal ultrasound can detect subtle structural abnormalities suggestive of low-grade inflammation, including bowel wall edema or thickening and mesenteric lymphadenopathy [27,28]. Although these findings are not disease-specific, when integrated with clinical and laboratory data, they may provide objective evidence of subclinical intestinal involvement [29].

To date, few studies have attempted to integrate food-specific IgG reactivity with objective markers of intestinal permeability, mucosal inflammation, microbiological alterations, and ultrasound abnormalities within a unified multimodal framework. Consequently, the possible existence of a multimodal clinical subgroup characterized by the coexistence of food-specific IgG reactivity, inflammatory biomarker abnormalities, and ultrasound findings remains insufficiently explored in pediatric populations.

Therefore, the aim of the present study was to explore associations between food-specific IgG reactivity patterns, inflammatory and permeability biomarkers, microbiological findings, and abdominal ultrasound abnormalities in children with chronic gastrointestinal symptoms. We hypothesized that children presenting increased food-specific IgG reactivity together with biomarker abnormalities and ultrasound changes may represent a clinically relevant subgroup characterized by convergent immune–gut alterations requiring further prospective investigation.

## 2. Materials and Methods

### 2.1. Study Design and Population

This retrospective pediatric study was conducted at Ponderas Academic Hospital, Bucharest, Romania, between January 2024 and January 2026. The study aimed to investigate the relationship between food-specific IgG reactivity, intestinal permeability, inflammatory biomarkers, microbiological findings, and ultrasound abnormalities in children with gastrointestinal symptoms.

A total of 552 pediatric patients aged 3–18 years were included and stratified into three predefined study groups:

1. Exploratory Immune-Activated Intestinal Profile (EIAIP) group (n = 196): children presenting chronic gastrointestinal symptoms associated with intestinal inflammatory activity, including elevated fecal calprotectin, increased zonulin, elevated fecal histamine, confirmed food-specific IgG polysensitization predominantly against gluten-containing cereals and dairy proteins, and ultrasound abnormalities suggestive of intestinal inflammation.
2. Classic symptomatic gastrointestinal group (n = 146): children presenting recurrent gastrointestinal symptoms without fulfilling the complete multimodal profile criteria.
3. Control group (n = 210): pediatric patients with normal abdominal ultrasound findings and inflammatory/permeability biomarkers, within laboratory reference ranges.

The study population included children referred to recurrent abdominal pain, bloating, altered bowel habits, food-related symptoms, nausea, constipation, diarrhea, or associated extra-intestinal manifestations such as fatigue or headaches. Because of the retrospective nature of the study, information regarding dietary intake, elimination diets, probiotic supplementation, previous antibiotic exposure, body mass index, nutritional status, and coexisting allergic diseases was not consistently available and therefore could not be incorporated into the statistical analyses.

Inclusion and Exclusion Criteria

The inclusion criteria were as follows: (1) age between 3 and 18 years; (2) availability of food-specific IgG testing performed as part of the clinical evaluation; (3) availability of abdominal ultrasound examination; (4) availability of intestinal inflammatory and permeability biomarkers, including fecal calprotectin, fecal histamine, and zonulin measurements; and (5) presence of recurrent gastrointestinal symptoms for at least 3 months, including abdominal pain, bloating, altered bowel habits, food-related gastrointestinal complaints, nausea, constipation, or diarrhea.

The exclusion criteria included (1) confirmed celiac disease; (2) inflammatory bowel disease previously established by endoscopic and histopathological evaluation; (3) known IgE-mediated food allergy; (4) severe chronic systemic disorders, including oncological, metabolic, genetic, or severe autoimmune diseases; (5) recent treatment with systemic corticosteroids, immunosuppressive agents, or biologic therapies; and (6) acute gastrointestinal infection at the time of presentation.

To minimize potential confounding factors, patients with conditions known to significantly influence intestinal inflammatory biomarkers, immune responses, or gastrointestinal ultrasound findings were excluded from the study.

All data were anonymized before analysis. Informed consent was obtained from parents or legal guardians, and assent was obtained from children when appropriate, in accordance with the Declaration of Helsinki. The study protocol was approved by the institutional ethics committee.

### 2.2. Food-Specific IgG Antibody Assessment

Food-specific IgG antibodies were assessed using a semi-quantitative multiplex immunoblot assay (MyFoodProfile^®^ 216 Foods, GA Generic Assays GmbH, Dahlewitz, Germany), allowing the simultaneous evaluation of IgG reactivity against 216 food antigens and food-related components. The panel included a broad spectrum of dietary antigens, comprising gluten-containing cereals (including wheat and gliadin), dairy proteins, egg white, egg yolk, soy, rice, corn, meat, fish, nuts, fruits, vegetables, and selected food additives incorporated within the commercial assay panel. Food extracts and individual food components were evaluated according to the manufacturer’s specifications, whereas processed foods were not analyzed as separate clinical categories. Results were expressed in arbitrary units per milliliter (AU/mL or U/mL) using the manufacturer’s calibration system. Although a common reporting scale was applied across the panel, the measurements reflected semi-quantitative antigen-specific immune reactivity and were not intended for direct comparison of absolute concentrations between different food antigens. Food-specific IgG responses were categorized according to predefined manufacturer thresholds, and binary positivity was determined using the corresponding assay cutoffs. Food-specific IgG testing was included as one component of a multimodal immune–gut assessment. In accordance with current international recommendations, food-specific IgG antibodies were not interpreted as diagnostic markers of food allergy, food intolerance, or clinically relevant food hypersensitivity. Instead, patterns of IgG reactivity were analyzed in relation to inflammatory biomarkers and abdominal ultrasound findings to investigate potential associations within the study population. Accordingly, no diagnostic or causal role was attributed to food-specific IgG antibodies in the study design, and IgG polysensitization was evaluated as an exploratory indicator of broader antigen exposure and immune recognition patterns. Laboratory analyses were performed in an ISO 15189:2022-accredited laboratory under standardized quality-controlled conditions. Fecal calprotectin and fecal histamine were measured in stool samples, whereas zonulin was measured in serum samples. All analyses were performed in an ISO 15189-accredited clinical laboratory using commercially available immunoassays according to the manufacturers’ instructions. Internal quality-control procedures were applied in accordance with laboratory standards. Results were expressed in arbitrary units per milliliter (AU/mL or U/mL), reflecting the intensity of antigen-specific immune reactivity.

IgG Reactivity Classification

Food-specific IgG responses were stratified into predefined categories:

- Class 0 (≤15 U/mL): no detectable reactivity;
- Class 1 (15–25 U/mL): low reactivity;
- Class 2 (25–50 U/mL): moderate reactivity;
- Class 3 (>50 U/mL): high-intensity reactivity.

For analytical purposes, IgG polysensitization was defined as reactivity ≥ 15 U/mL to at least five food antigens, and high immune burden was defined as reactivity to ≥10 food antigens. The threshold of five positive food antigens was selected as an exploratory operational cutoff intended to characterize broader immune-reactivity patterns and should not be interpreted as a validated clinical definition of food hypersensitivity.

Particular attention was given to the most frequently observed food-specific IgG reactivity patterns, including reactivity to gluten-containing cereals, dairy proteins, and combined gluten–dairy reactivity. Combined gluten–dairy reactivity was defined as the coexistence of positive IgG responses to at least one gluten-containing cereal (or gluten-related component) and at least one dairy protein in the same individual. This category represented an analytical subgroup and not a distinct laboratory measurement.

Rather than focusing on individual food antigens, the analyses examined broader patterns of food-specific IgG reactivity across multiple dietary categories. This approach was intended to evaluate potential associations between cumulative antigen exposure patterns, inflammatory and permeability biomarkers, microbiological findings, and abdominal ultrasound abnormalities within the study population.

### 2.3. Intestinal Inflammatory and Permeability Biomarkers

The inflammatory and intestinal barrier profiles of the study population were assessed using faecal calprotectin, zonulin, faecal histamine, and microbiological stool testing. Calprotectin served as a marker of intestinal inflammation, while zonulin was evaluated as an exploratory marker of barrier function. Due to concerns regarding the specificity and validity of commercial zonulin assays, zonulin results were interpreted cautiously and considered supportive rather than definitive evidence of altered permeability. Faecal histamine indicated mast-cell-associated mucosal activity. Stool microbiology included testing for Dientamoeba fragilis and other relevant enteric pathogens when indicated. All results were interpreted using pediatric laboratory reference ranges and integrated with clinical and ultrasound data to explore potential immune–gut associations.

Biomarker elevations were interpreted using laboratory-specific pediatric reference ranges.

### 2.4. Abdominal Ultrasound Assessment

Abdominal ultrasound examinations were performed using a high-resolution ultrasound system (ACUSON). Abdominal ultrasound examinations were performed using a high-resolution system (ACUSON NX3 Elite, Siemens Healthineers, Erlangen, Germany) with broadband, multifrequency transducers optimised for pediatric imaging.

- High-frequency linear transducer (5–12 MHz) for detailed assessment of bowel wall layers and mesenteric lymph nodes

All examinations followed standardised pediatric ultrasonography protocols. Gain, focal zones, depth, and frequency were dynamically adjusted to optimise image quality based on patient age and body habits. The imaging protocol included Tissue Harmonic Imaging (THI), Speckle Reduction Imaging (SRI), adaptive grayscale optimisation, and Colour and Power Doppler evaluation when required. Scanning was performed systematically in longitudinal and transverse planes, with additional oblique sections as needed. Intestinal wall thickness was measured at the terminal ileum and colon using integrated electronic callipers.

Ultrasound Definitions

- Bowel wall thickening/edema: >3 mm,
- Mesenteric lymphadenopathy: lymph nodes > 8 mm short-axis diameter.

All ultrasound examinations were performed by a single experienced pediatric physician with certified ultrasonography competence, ensuring methodological consistency and minimizing inter-observer variability. Abdominal ultrasound examinations were performed by a single experienced pediatric physician with certified ultrasonography competence using a standardized examination protocol. The use of a single operator minimized inter-observer variability; however, formal reproducibility analyses, including intra-observer and inter-observer agreement assessments, were not performed and represent a study limitation.

### 2.5. Clinical Symptom Assessment

Clinical manifestations were systematically recorded using structured parental questionnaires and physician-supervised interviews.

The evaluated symptoms included recurrent abdominal pain, bloating, constipation, diarrhea, nausea, altered bowel habits, fatigue, headaches, and extra-intestinal manifestations.

Symptom severity was graded using a 4-point Likert-type scale:

- 0 = absent;
- 1 = mild;
- 2 = moderate;
- 3 = severe.

A cumulative symptom burden score was additionally calculated for correlation analyses.

### 2.6. Statistical Analysis

Statistical analyses were performed using GraphPad Prism version 9.5.1 (GraphPad Software, San Diego, CA, USA). A structured multilevel analytical framework was applied to investigate associations between immunological, inflammatory, microbiological, clinical, and imaging parameters. The distribution of continuous variables was assessed using the Shapiro–Wilk test. Normally distributed variables were expressed as mean ± standard deviation (SD) and compared using Student’s *t*-test or one-way ANOVA. Non-normally distributed variables were expressed as median and interquartile range (IQR) and analyzed using Mann–Whitney U or Kruskal–Wallis tests. Categorical variables were reported as frequencies and percentages and compared using Chi-square or Fisher’s exact tests. Correlation analyses between food-specific IgG burden, biomarker concentration, ultrasound abnormalities, and clinical symptom scores were performed using Pearson or Spearman correlation coefficients, depending on data distribution. Odds ratios (ORs) with corresponding 95% confidence intervals (CIs) were calculated to quantify associations between inflammatory biomarkers and ultrasound abnormalities. Multivariable logistic regression models were constructed to identify independent predictors of pathological ultrasound findings, adjusting for potential confounders, including age and sex. Receiver operating characteristic (ROC) curve analysis was additionally performed to evaluate the classification performance of isolated biomarkers and integrated multimodal models. To reduce the risk of type I error associated with multiple comparisons, Bonferroni correction was applied where appropriate. All statistical tests were two-sided, and a *p*-value < 0.05 was considered statistically significant.

Parental consent and child assent (when appropriate) were obtained in accordance with the Declaration of Helsinki. Our study protocol was approved by the institutional ethics committee of Ponderas Academic Hospital (Protocol No 296/2 May 2026).

## 3. Results

### 3.1. Demographic, Clinical, and Biomarker Characteristics of the Study Population

**Figure 1:**
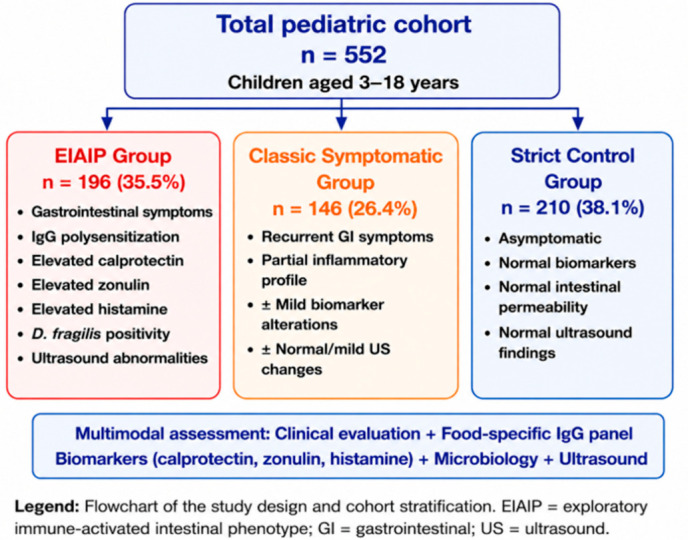
Study flowchart illustrating cohort stratification and multimodal characterization of pediatric patients included in the study. The exploratory immune-activated intestinal phenotype (EIAIP) group represented a subgroup characterized by the co-occurrence of gastrointestinal symptoms, food-specific IgG polysensitization, biomarker abnormalities, microbiological findings, and ultrasound changes.

A total of 552 pediatric patients aged 3–18 years were included in the final study cohort and stratified into three predefined groups: an exploratory immune–gut inflammatory profile group (EIAIP) group comprising 196 children (35.5%), a classic symptomatic gastrointestinal group including 146 children (26.4%), and a strict control group consisting of 210 children (38.1%); see Figure 1. The overall cohort demonstrated a slight male predominance, with boys representing approximately 56% of participants. Mean age distributions were comparable across groups, with no statistically significant differences in age or sex distributions (*p* > 0.05), ensuring baseline comparability.

Children included in the EIAIP cohort presented the highest clinical burden and demonstrated a characteristic multimodal inflammatory profile, including elevated fecal calprotectin, increased zonulin, elevated fecal histamine, food-specific IgG polysensitization, and pathological ultrasound findings suggestive of intestinal inflammation.

The classic symptomatic group included children with recurrent gastrointestinal manifestations without fulfilling the complete multimodal profile criteria. In contrast, the strict control group showed normal inflammatory biomarker levels, normal intestinal permeability markers, and normal abdominal ultrasound findings.

The most frequently reported clinical manifestations in symptomatic patients included recurrent abdominal pain, bloating, altered bowel habits, diarrhea, constipation, nausea, and fatigue. Extra-intestinal manifestations, including headaches, fatigue, and dermatological symptoms, were significantly more frequent in the EIAIP group, supporting the concept of systemic immune-mediated involvement.

**Figure 2:**
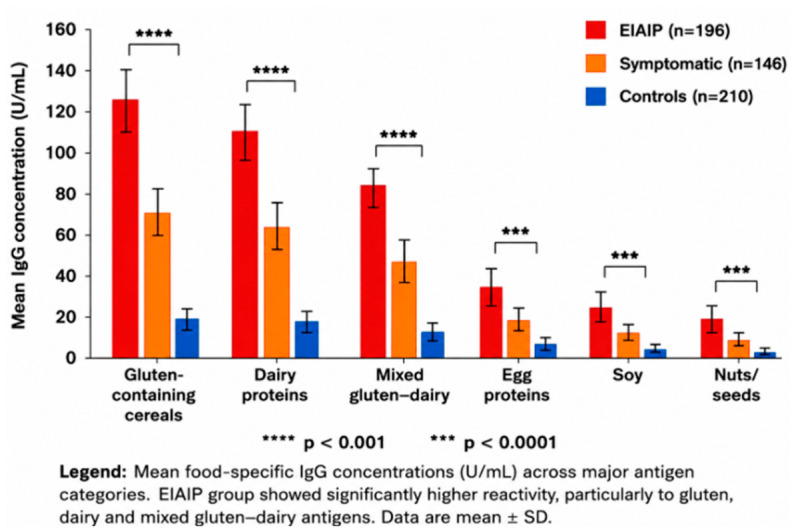
Comparison of food-specific IgG reactivity patterns across major antigen categories in the three study groups. Children within the exploratory immune–gut inflammatory profile group demonstrated significantly higher IgG responses against gluten-containing cereals, dairy proteins, and mixed gluten–dairy antigens compared with both symptomatic children and strict controls. Data are presented as mean ± SD. Statistical significance is indicated directly on the graph.

Food-specific IgG polysensitization was markedly more prevalent in the EIAIP cohort, particularly involving gluten-containing cereals, dairy proteins, and mixed gluten–dairy reactivity patterns. Elevated inflammatory and permeability biomarkers were also significantly enriched in this group compared with both classic symptomatic children and strict controls; see Figure 2.

Collectively, these findings identify a subgroup characterized by the co-occurrence of gastrointestinal symptoms, food-specific IgG polysensitization, inflammatory biomarker abnormalities, and ultrasound changes; see Table 1.

### 3.2. Ultrasound Findings and Their Relationship with Immune Activation

Ultrasound abnormalities were significantly more frequent in children belonging to the EIAIP cohort compared with both classic symptomatic children and strict controls. Bowel wall edema/thickening (>3 mm) represented the predominant imaging abnormality and was mainly localized at the level of the terminal ileum and right colon. Mesenteric lymphadenopathy, previously less prominent in smaller cohorts, became highly significant in the expanded inflammatory phenotype group, suggesting broader systemic symptom burden within this subgroup. Doppler evaluation additionally demonstrated increased vascularization in a subset of EIAIP patients, further suggesting changes potentially compatible with inflammatory activity. In contrast, strict controls demonstrated preserved bowel wall morphology, absence of significant mesenteric lymphadenopathy, and normal vascular patterns; see Table 2.

### 3.3. Correlation Analysis Between Immune Markers, Inflammatory Biomarkers, and Ultrasound Findings

**Figure 3:**
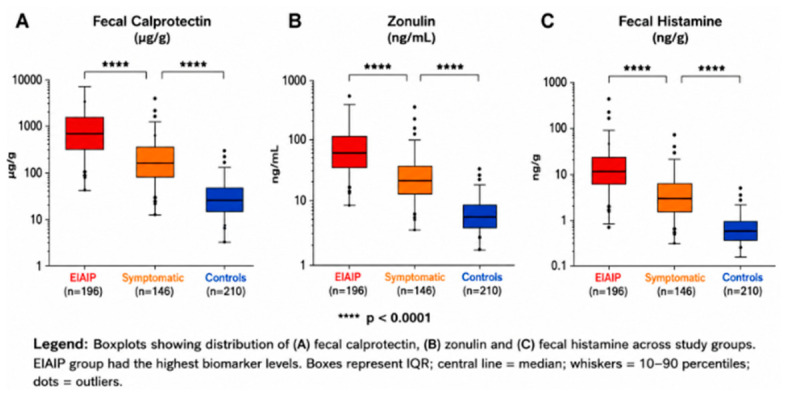
Distribution of intestinal inflammatory and permeability biomarkers across study groups. Boxplots demonstrate significantly elevated fecal calprotectin, zonulin, and fecal histamine concentration in children with exploratory immune-activated intestinal phenotype compared with symptomatic children and strict controls. The EIAIP cohort demonstrated the highest inflammatory burden and widest biomarker variability, demonstrating greater biomarker variability within this subgroup.

**Figure 4:**
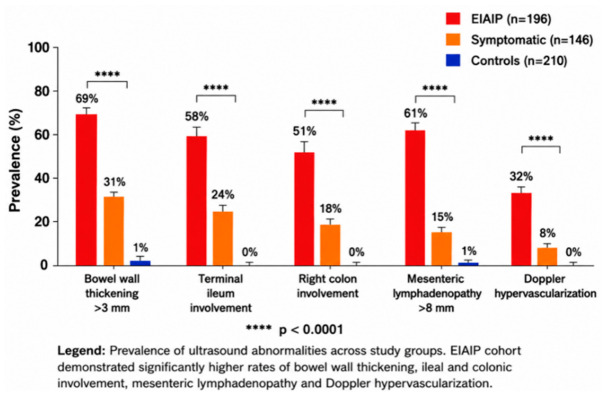
Distribution of ultrasound abnormalities among study groups. Children with immune-activated intestinal phenotypes exhibited significantly higher prevalence of bowel wall edema/thickening, terminal ileum involvement, mesenteric lymphadenopathy, and Doppler hypervascularization compared with symptomatic children and controls. These findings indicate a higher prevalence of ultrasound abnormalities within this subgroup.

Correlation analyses demonstrate moderate-to-strong associations between food-specific IgG burden, intestinal permeability markers, inflammatory biomarkers, and ultrasound abnormalities; see Table 3. A moderate positive correlation was observed between total IgG burden and bowel wall thickness, suggesting that higher cumulative IgG reactivity was associated with more pronounced structural intestinal changes. Fecal calprotectin demonstrated the strongest association with ultrasound inflammatory abnormalities, while zonulin correlated significantly with both IgG burden and inflammatory biomarkers, suggesting a potential association between epithelial barrier alterations and immune–gut interactions. Elevated fecal histamine concentration additionally correlated with symptom severity, particularly abdominal pain and bloating, suggesting possible mast-cell-associated biological activity; see Figure 3 and Figure 4.

### 3.4. Multivariable Logistic Regression Analysis

Multivariable logistic regression analysis identified several independent predictors of pathological ultrasound findings, including bowel wall edema/thickening and mesenteric lymphadenopathy.

**Figure 5:**
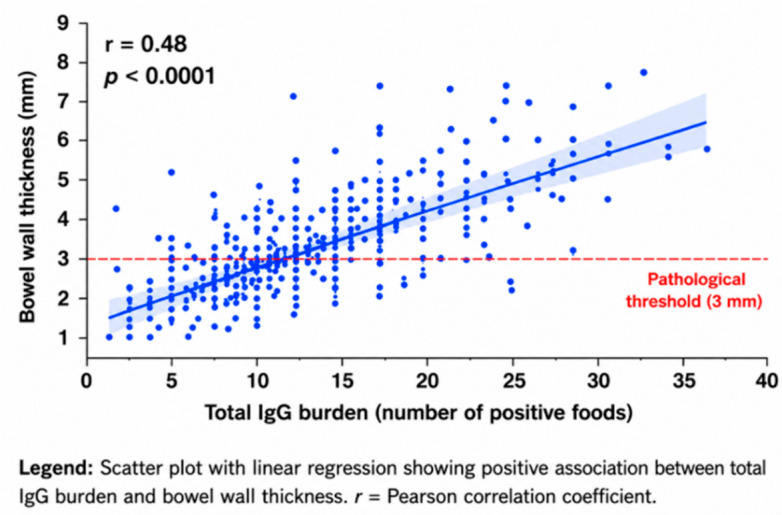
Scatter plot demonstrating the relationship between cumulative food-specific IgG burden and bowel wall thickness assessed by abdominal ultrasound in participants from all study groups, including healthy controls, children with classic gastrointestinal symptoms, and children classified within the exploratory immune–gut inflammatory profile group. Increasing IgG reactivity was associated with progressively greater intestinal wall thickening, supporting a potential relationship between chronic immune activation and structural intestinal abnormalities. The solid line represents the linear regression model with the shaded area indicating the 95% confidence interval. A significant positive correlation was observed between total IgG burden and bowel wall thickness (Pearson r = 0.48, *p* < 0.0001).

A significant positive correlation was identified between cumulative food-specific IgG burden and bowel wall thickness measured by abdominal ultrasound. Children presenting higher numbers of positive food-specific IgG reactions demonstrated progressively increased intestinal wall thickness, suggesting an association between chronic immune exposure and structural intestinal involvement. Scatter plot analysis revealed a moderate positive correlation between total IgG burden and bowel wall thickness (r = 0.48, *p* < 0.0001). The relationship remained significant across the entire pediatric cohort and was particularly pronounced in children belonging to the exploratory immune-activated intestinal phenotype (EIAIP) group; see Figure 5.

The strongest predictors were elevated calprotectin and zonulin concentration, followed by mixed dairy–gluten IgG reactivity and high IgG polysensitization burden (≥10 foods). Elevated fecal histamine and Dientamoeba fragilis positivity also remained independently associated with ultrasound abnormalities after adjustment for age and sex; see Table 4.

**Figure 6:**
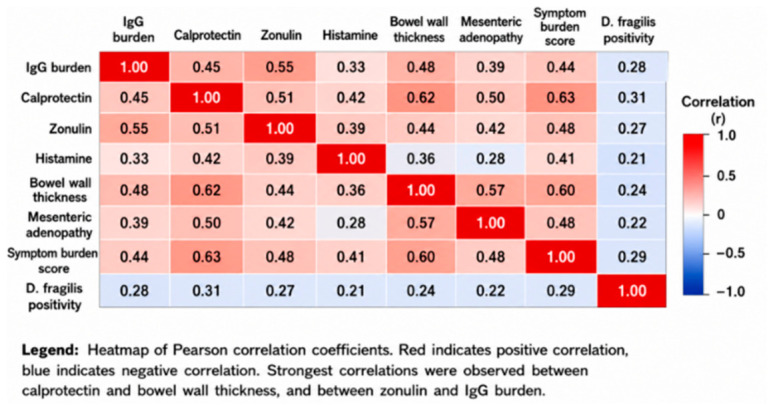
Correlation heatmap illustrating relationships between immune activation markers, intestinal permeability biomarkers, inflammatory parameters, ultrasound abnormalities, and clinical symptom burden. Strong positive correlations were identified between calprotectin and bowel wall thickening, as well as between zonulin and cumulative IgG burden, supporting potential interactions between immune-related biomarkers and ultrasound findings.

To further investigate the interactions between immune activation, intestinal permeability, inflammatory biomarkers, microbiological findings, and structural intestinal abnormalities, a comprehensive correlation matrix analysis was performed across the entire pediatric cohort. The strongest positive correlation was identified between fecal calprotectin concentration and bowel wall thickness (r = 0.62, *p* < 0.0001), supporting the role of mucosal inflammation in the development of ultrasound-detected intestinal abnormalities. Significant moderate correlations were additionally observed between zonulin and cumulative IgG burden (r = 0.55, *p* < 0.0001), suggesting a close relationship between intestinal permeability dysfunction and chronic antigen exposure; see Figure 6.

### 3.5. Receiver Operating Characteristic (ROC) Analysis

Receiver operating characteristic (ROC) curve analysis was performed to evaluate the classification performance of isolated biomarkers and integrated multimodal models in identifying children with pathological intestinal ultrasound findings. Food-specific IgG reactivity alone demonstrated moderate discriminative ability, while ultrasound findings and calprotectin exhibited higher classification performance. The integrated multimodal model combining IgG burden, calprotectin, zonulin, histamine, microbiological findings, and ultrasound abnormalities achieved the highest classification performance. Pairwise comparison of ROC curves using DeLong’s test demonstrated statistically significant superiority of the multimodal model compared with isolated biomarkers; see Table 5.

Food-specific IgG reactivity alone demonstrated moderate discriminative ability, with an area under the curve (AUC) of 0.79, indicating limited performance when interpreted as an isolated biomarker. Ultrasound findings alone showed improved classification accuracy (AUC = 0.83), while fecal calprotectin demonstrated the strongest performance among individual biomarkers (AUC = 0.86), reflecting its close association with mucosal inflammatory activity.

**Figure 7:**
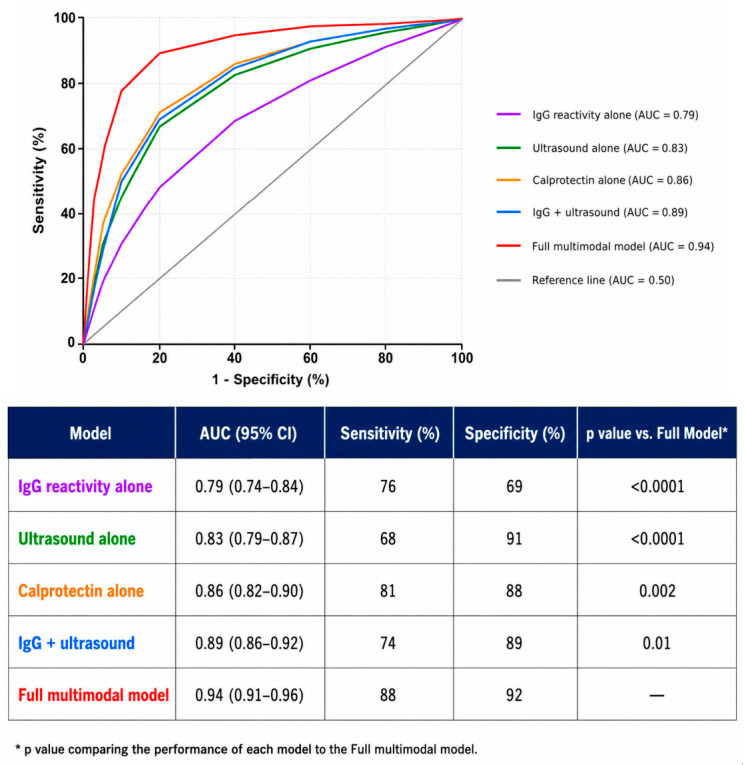
Receiver operating characteristic (ROC) curve analysis demonstrating the classification performance of isolated biomarkers and integrated multimodal models. The full multimodal model combining food-specific IgG burden, calprotectin, zonulin, histamine, microbiological findings, and ultrasound abnormalities achieved the highest diagnostic accuracy, significantly outperforming isolated biomarkers.

Combining food-specific IgG burden with ultrasound abnormalities significantly improved classification performance (AUC = 0.89), supporting the value of integrated serological and imaging assessment. However, the highest diagnostic accuracy was achieved by the full multimodal model integrating food-specific IgG burden, calprotectin, zonulin, fecal histamine, microbiological findings, and ultrasound abnormalities, which demonstrated excellent discriminative performance (AUC = 0.94); see Figure 7.

## 4. Discussion

### 4.1. Integrated Immune–Gut Assessment and Identification of an Exploratory Clinical Subgroup

Functional gastrointestinal disorders (FGIDs) and chronic food-related gastrointestinal symptoms in children are increasingly recognized as multifactorial conditions involving complex interactions among the intestinal barrier, mucosal immune responses, microbiota composition, dietary exposures, and neuroimmune pathways [20,21,22]. Within this context, the present study applied an integrated multimodal approach combining food-specific IgG reactivity patterns, inflammatory and barrier-related biomarkers, microbiological findings, and abdominal ultrasound data to characterize children presenting chronic gastrointestinal symptoms.

Rather than identifying a validated clinical phenotype, our analysis revealed a subgroup of symptomatic children characterized by the co-occurrence of gastrointestinal symptoms, food-specific IgG polysensitization, biomarker abnormalities, microbiological findings, and ultrasound changes. Compared with the remaining study population, these children more frequently exhibited elevated calprotectin concentrations, increased zonulin values, higher fecal histamine concentrations, and a greater prevalence of bowel wall thickening and mesenteric lymphadenopathy on ultrasound examination [23,24,25,26,27].

The observed associations between cumulative food-specific IgG reactivity, inflammatory biomarkers, and ultrasound findings suggest the existence of complex interactions between dietary antigen exposure, immune-related responses, intestinal barrier function, and gastrointestinal symptoms. However, given the observational design of the study and the absence of histopathological confirmation, these findings should be interpreted as associations rather than evidence of causal relationships or confirmed intestinal inflammation [27,28,29].

The clinical applicability of the proposed multimodal framework remains uncertain. The present model was developed as an exploratory research tool and should not be considered a substitute for established pediatric gastrointestinal diagnostic approaches, including clinical evaluation, laboratory testing, endoscopy, histopathological assessment, and disease-specific diagnostic criteria. Its potential value may reside in identifying subgroups of symptomatic children presenting convergent biomarkers and imaging abnormalities that warrant further investigation. However, prospective validation studies directly comparing this approach with current diagnostic strategies are required before any clinical implementation can be recommended [30,31,32,33,34,35].

Collectively, the results support the concept that multimodal assessment may identify clinically relevant subgroups among children with chronic gastrointestinal symptoms. Further prospective studies incorporating longitudinal follow-up, mechanistic investigations, and histological validation are required to determine the biological and clinical significance of these observations [33,34,35,36].

### 4.2. Clinical Significance of Food-Specific IgG Reactivity

The clinical significance of food-specific IgG antibodies remains a subject of ongoing debate in both allergy and gastroenterology research [23,24]. Current international position statements consider food-specific IgG antibodies to reflect dietary antigen exposure and immune recognition rather than clinically relevant food hypersensitivity. Consequently, food-specific IgG testing is not recommended as a standalone diagnostic tool for food allergy or food intolerance [37,38,39,40].

Our findings should be interpreted within this framework. Children presenting broader patterns of food-specific IgG reactivity more frequently exhibited inflammatory biomarker abnormalities and ultrasound changes than controls. Furthermore, cumulative IgG burden showed significant associations with bowel wall thickness and mesenteric lymphadenopathy. These observations do not establish a pathogenic role for food-specific IgG antibodies but suggest that broader IgG-reactivity patterns may coexist with other biological markers in a subset of symptomatic pediatric patients [41,42].

Emerging evidence from recent clinical studies suggests that food-specific IgG reactivity may be associated with inflammatory pathways in selected populations, particularly when evaluated within a broader immune–gut context rather than as an isolated diagnostic marker [43,44,45,46,47,48,49]. Accordingly, the present findings support further investigation of food-specific IgG patterns as potential contextual biomarkers of immune–diet interactions while emphasizing that their diagnostic and clinical utility remains to be established [50,51,52,53,54].

### 4.3. Study Limitations

Several limitations should be considered when interpreting the present findings. First, the retrospective observational design precludes causal inference and limits the ability to determine temporal relationships between food-specific IgG reactivity, biomarker abnormalities, and ultrasound findings. Second, no endoscopic or histopathological confirmation was available, preventing direct verification of intestinal inflammatory changes. Third, information regarding dietary habits, elimination diets, body mass index, probiotic supplementation, antibiotic exposure, and allergic comorbidities was not consistently available and therefore could not be incorporated into the analyses. Fourth, the analytical specificity and biological validity of commercially available zonulin assays remain subjects of ongoing debate. Due to the retrospective design, detailed assay-specific validation parameters (e.g., analytical sensitivity, intra-assay and inter-assay coefficients of variation) were not available for inclusion. Fifth, incorporation bias may have occurred because several variables used to characterize the study subgroups were subsequently included in comparative analyses and predictive models, potentially contributing to overestimation of classification performance. Finally, the proposed multimodal framework has not undergone external validation, cross-validation, or bootstrap validation and should therefore be regarded as exploratory and hypothesis-generating.

Because all ultrasound examinations were performed by a single operator, formal assessment of intra-observer reproducibility and inter-observer agreement was not available. Although a standardized examination protocol was applied throughout the study, the reproducibility of ultrasound measurements could not be formally evaluated.

## 5. Conclusions

Our findings identify a subgroup of children with chronic gastrointestinal symptoms characterized by the co-occurrence of food-specific IgG polysensitization, inflammatory biomarker abnormalities, and ultrasound changes. The observed associations support further investigation of integrated immune–gut assessment strategies, but should be considered exploratory and hypothesis-generating. Food-specific IgG testing should not be interpreted as a standalone diagnostic tool, and prospective studies with independent validation cohorts are required to determine the clinical significance of these observations.

## Figures and Tables

**Table 1:** Comparison of demographic characteristics, gastrointestinal manifestations, food-specific IgG reactivity patterns, and inflammatory/permeability biomarkers across study groups. The EIAIP cohort demonstrated the highest burden of multisystem symptoms, IgG polysensitization, inflammatory biomarkers, and biomarker abnormalities potentially associated with intestinal barrier dysfunction.

Variable	IAIP (n = 196)	Symptomatic (n = 146)	Controls (n = 210)	*p*-Value
Age (years), mean ± SD	9.8 ± 4.1	9.3 ± 4.0	9.5 ± 3.9	NS
Male sex (%)	56%	56%	55%	NS
Abdominal pain (%)	91%	74%	8%	<0.01
Bloating (%)	82%	61%	5%	<0.005
Altered bowel habits (%)	76%	49%	4%	<0.01
Nausea/Vomiting (%)	48%	31%	3%	<0.01
Fatigue (%)	58%	29%	3%	<0.0001
Dermatological symptoms (%)	37%	18%	2%	<0.0001
IgG ≥ 5 foods (%)	88%	76%	24%	<0.0001
IgG ≥ 10 foods (%)	65%	42%	8%	<0.01
Dairy IgG reactivity (%)	78%	62%	18%	<0.0001
Gluten IgG reactivity (%)	70%	53%	15%	<0.01
Mixed dairy–gluten reactivity (%)	58%	38%	7%	<0.0001
Elevated calprotectin (%)	74%	31%	2%	<0.0001
Elevated zonulin (%)	69%	28%	2%	<0.0001
Elevated fecal histamine (%)	54%	19%	1%	<0.0001
*Dientamoeba fragilis* positivity (%)	28%	9%	1%	<0.0001

**Table 2:** Distribution of ultrasound abnormalities across study groups. Children with an immune-activated intestinal phenotype demonstrated significantly higher prevalence of bowel wall edema/thickening, terminal ileum involvement, mesenteric lymphadenopathy, and Doppler hypervascularization, representing ultrasound findings potentially associated with intestinal inflammatory activity.

Ultrasound Finding	IAIP (n = 196)	Symptomatic (n = 146)	Controls (n = 210)	*p*-Value
Bowel wall edema/thickening > 3 mm (%)	69%	31%	1%	<0.0001
Terminal Ileum involvement (%)	58%	24%	0%	<0.01
Right colon involvement (%)	51%	18%	0%	<0.01
Mesenteric lymphadenopathy > 8 mm (%)	61%	15%	1%	<0.0001
Doppler hypervascularization (%)	32%	8%	0%	<0.0001

**Table 3:** Correlation analysis demonstrates significant relationships between immune activation, intestinal permeability, inflammatory biomarkers, ultrasound abnormalities, and clinical symptom burden. The strongest association was observed between fecal calprotectin and bowel wall inflammatory changes.

Variables Compared	Correlation (r)	*p*-Value	Interpretation
IgG burden vs. bowel wall thickness	0.48	<0.0001	Moderate positive correlation
Calprotectin vs. bowel wall thickness	0.62	<0.0001	Strong positive correlation
Zonulin vs. IgG burden	0.55	<0.0001	Moderate–strong correlation
Zonulin vs. calprotectin	0.51	<0.0001	Moderate positive correlation
Histamine vs. abdominal pain severity	0.39	<0.001	Moderate correlation
*D. fragilis* positivity vs. calprotectin	0.31	<0.01	Weak–moderate correlation
Mesenteric adenopathy vs. calprotectin	0.50	<0.0001	Moderate positive correlation

**Table 4:** Multivariable logistic regression analysis demonstrates independent predictors of pathological ultrasound findings. Elevated calprotectin and zonulin demonstrated the strongest associations with pathological ultrasound findings.

Predictor	Adjusted OR	95% CI	*p*-Value
IgG ≥ 10 foods	4.9	2.9–8.6	<0.0001
Mixed dairy–gluten IgG reactivity	5.4	3.1–9.3	<0.0001
Elevated calprotectin	7.8	4.5–13.4	<0.0001
Elevated zonulin	6.5	3.8–11.1	<0.0001
Elevated fecal histamine	3.2	1.7–6.0	0.001
*D. fragilis* positivity	3.7	1.8–7.4	<0.001

**Table 5:** Receiver operating characteristic analysis demonstrating the classification performance of isolated biomarkers and integrated multimodal models. The full multimodal model combining immunological, inflammatory, permeability, microbiological, and imaging parameters achieved the highest diagnostic accuracy. Because several variables included in the multimodal model were also used in the definition of the study subgroups, the reported classification performance may be overestimated and should be interpreted as exploratory. External validation in independent cohorts will be required before any clinical application can be considered.

Model	AUC (95% CI)	Sensitivity	Specificity
IgG reactivity alone	0.79	76%	69%
Ultrasound alone	0.83	68%	91%
Calprotectin alone	0.86	81%	88%
IgG + ultrasound	0.89	74%	89%
Full multimodal model	0.94	88%	92%

## Data Availability

The datasets generated and analyzed during the current study are available from the corresponding author upon request.
